# Who suffered most after deaths due to COVID-19? Prevalence and correlates of prolonged grief disorder in COVID-19 related bereaved adults

**DOI:** 10.1186/s12992-021-00669-5

**Published:** 2021-02-11

**Authors:** Suqin Tang, Zhendong Xiang

**Affiliations:** 1 Department of Sociology, Law School, Shenzhen University, L3–1217, Zhili Building, Canghai Campus, Shenzhen, 618010 China; 2 Shenzhen Yishi Huolala Technology Limited Company, Futian District, Shenzhen, 518049 China

**Keywords:** Bereavement, Prolonged grief disorder, Persistent complex bereavement disorder, Prevalence, COVID-19, China

## Abstract

**Background:**

Deaths by COVID-19 have left behind nearly 12 million recent bereaved individuals worldwide and researchers have raised concerns that the circumstances of COVID-19 related deaths will lead to a rise prevalence of prolonged grief disorder (PGD) cases. However, to date, no studies have examined the prevalence of PGD among people bereaved due to COVID-19. This study aimed to estimate the prevalence of PGD and investigated demographic and loss-related factors associated with prolonged grief symptoms among Chinese individuals bereaved due to COVID-19.

**Methods:**

This was a cross-sectional online survey conducted between September 1 and October 3, 2020. A total of 422 Chinese participants (55.5% males; 32.73 [9.31] years old) who lost a close person due to COVID-19 participated in the study. Demographic and loss-related information was collected, and self-reported prolonged grief symptoms were measured by a 13-item International Prolonged Grief Disorder Scale (IPGDS: 1–65) and a 17-item Traumatic Grief Inventory Self Report (TGI-SR: 1–85). Multiple linear regression analysis was used to determine the associated factors of levels of grief symptoms.

**Results:**

Prevalence of PGD was 37.8% screened by IPGDS and 29.3% by TGI-SR. No difference was found in levels of grief symptoms between participants whose close one died more than 6 months ago and those who experienced the loss less than 6 months ago. More severe prolonged grief symptoms assessed by IPGDS was associated with losing a close person by COVID-19 rather than complications (B: 5.35; 95% CI: 0.54–10.05), losing a partner (B: 7.80; 95% CI: 3.24–12.37), child (B: 8.15; 95% CI: 1.03–15.26), and parent (B: 5.49; 95% CI: 1.49–9.48) rather than losing a relative or a person with other relationship, feeling more traumatic about the loss (B: 1.71; 95% CI: 0.52–2.90), being closer with the deceased (B: 1.60; 95% CI: 0.34–2.86). Moreover, Losing a grandparent (B: 6.62; 95% CI: 0.53–12.71) and having more conflicts with the deceased (B: 1.05; 95% CI: − 0.008–2.11) were related to higher levels of grief symptoms assessed by TGI-SR.

**Conclusions:**

Echoing researchers’ concerns, the prevalence of PGD is high among people bereaved due to COVID-19. Individuals with a higher risk of developing PGD should be identified and bereavement support should be offered as early as possible.

## Background

By January 8, 2021, there have been over 86 million confirmed cases of coronavirus disease (COVID-19), including more than 1.8 million deaths [1]. As shown in a recent study, for each COVID-19 death, about 9 family members would be affected and grieve [2]. It is estimated that the mass bereavement due to COVID-19 will leave behind nearly 16 million newly bereaved people globally. If deaths of close friends were counted, this number could be even larger. Given the lasting impacts on the bereaved population brought by the COVID-19 pandemic, delivering efficient care for people who bereaved due to COVID-19 becomes a worldwide challenge.

After a close person died, 9.8% of people would develop prolonged grief disorder (PGD) [3], a newly added diagnosis in the International Classification of Diseases eleventh edition (ICD-11) [4]. PGD is a persistent and pervasive grief response characterized by longing for the deceased and/or persistent preoccupation with the deceased, accompanied by intense emotional pain including sadness, guilt, anger, denial, blame, difficulty accepting the death, feeling one has lost a part of one‘s self, an ability to experience positive mood and so on. This grief response has persisted for an atypically long period of time following the loss (more than 6 months at a minimum) and clearly exceeds expected social, cultural, or religious norms for the individual’s culture and context, and results in significant impairment in personal, family, occupational and other important areas of functioning. The Diagnostical and Statistical Manual of Mental Disorders fifth edition (DSM-5) published in 2013 included Persistent Complex Bereavement Disorder in Section 3 under Conditions for Further Study [5] and is considered to be replaced by a diagnosis also named prolonged grief disorder in Section 2 under Diagnostic Criteria and Codes in the forthcoming version of the DSM-5 [6, 7].

Researchers have raised concerns that the circumstances of COVID-19 related deaths will lead to a worldwide elevated prevalence of PGD cases [8, 9]. First, COVID-19 related deaths are usually unexpected, and the unexpectedness of death yielded higher levels of prolonged grief symptoms in Australian [10], Dutch [11], Japanese [12], Chinese [13], and Thai [14] bereaved individuals. Second, COVID-19 related deaths could be traumatic as people may experience the pandemic as a disaster. Survivors of unnatural deaths like disasters and accidents are at a higher level of developing prolonged grief [15, 16]. The traumatic nature of COVID-19 related deaths is also manifested by being unable to visit the dying person and hold and attend traditional funerals and other rituals. A study revealed that among bereaved whose family members died in ICU, the likelihood of developing prolonged grief for those who did not have the chance to say goodbye to the deceased person was 2 to 3 times to other bereaved individuals [17]. As the subjective experience of traumatic death increases, the severity of prolonged grief symptoms increases [18].

Despite the consensus that mental health professionals should pay attention to the rise of prolonged grief disorder after the COVID-19 pandemic [8, 9] and call for evidence-based and culturally sensitive bereavement care for individuals bereaved during the pandemic [19–24], no study specifically focused on bereaved people and the impact that a death linked to a pandemic had on their subsequent grief before the outbreak of the COVID-19 pandemic [25]. After the COVID-19 outbreak, one empirical study included 49 Dutch individuals who experienced COVID-19 related bereavement showed that they reported more severe grief than people who experienced natural losses and equivalent levels of grief with people who experienced unnatural losses [26]. Other than that, little is known about the prevalence and symptom severity of prolonged grief disorder among people whose close ones died from the COVID-19 pandemic.

In order to identify at-risk bereaved people who experienced deaths due to COVID-19, a number of loss-related factors and their association with the prolonged grief symptom severity should be examined. First, previous studies have demonstrated that closer kinship was related to higher levels of prolonged grief symptoms across age groups, causes of death, and cultures [13, 16, 27–35]. In addition to the objective nature of the relationship between the deceased and the bereaved, the quality of their relationship matters. Quality of mourner-decedent relationship contains two salient factors, namely closeness and conflict with the deceased [36]. According to the attachment-based model, losing a close person represents the loss of an important attachment figure, and the more deeply one was attached to the deceased person, the more difficult it for him/her to integrate the reality of death and update the mental representation of the deceased [37]. On the other hand, clinical observations showed that conflicts exist at the time of death and earlier may lead to pathological mourning [38]. Hence, it is worthwhile to investigate the role of the closeness and conflict with the deceased in relation to prolonged grief. Last, as mentioned earlier, unexpected death [10–14] and losses with traumatic features [15–18] tended to yield more severe prolonged grief symptoms.

Therefore, this study aimed to examine the prevalence and severity of prolonged grief symptoms and to investigate demographic and loss-related factors associated with prolonged grief symptoms among individuals bereaved due to COVID-19. We hypothesized that a) the prevalence and severity of prolonged grief would be high, b) closer kinship with the deceased would be related to more severe symptoms, c) higher levels of unexpectedness and traumatic experience of the death/loss would be correlated with higher grief symptom levels, and d) both closeness and conflict with the deceased would be related to grief symptoms levels.

## Methods

### Participants and procedure

Data collection was conducted from September 1, 2020, to October 3, 2020. To be eligible to participate in the study, participants had to be 18 and/or above years old and lose a close one due to COVID-19. Participants were recruited through social network websites (e.g., Baidu, WeiBo) and mobile applications (e.g., WeChat). A brief introduction of the study and inclusion criteria for participants were stated, and a link to the online survey was posted in the recruiting advertisement. Once the participants accessed the online survey via the link, the consent page including the purpose of the study, voluntariness of participation, confidentiality, and grief support resources would be presented before entering the formal survey. Only when the participants ticked the box “I understand the information described above and agree to participate in this study”, they would enter the formal survey. At the end of the survey, support resources specifically for bereaved people during the COVID-19 were listed, including free counseling and grief counseling information, self-help resources, and books. Ethical approval was obtained before data collection from the Ethics Committee of the Health Science Center, Shenzhen University.

A sample of 476 participants submitted the questionnaire, and 54 of whom were excluded from the analysis due to response time less than 5 min (*n* = 21), inconsistent information about the deceased person (*n* = 15), bereaved more than 9 months ago (*n* = 11), and patterned responses (*n* = 8). Therefore, data of 422 participants were analyzed.

### Measures

#### Demographic and loss-related information

Demographics included sex, age, education, and religious belief. Loss-related information included relationship to deceased (i.e., partner, child, parent, grandparent, relative, friend, other relationship), age of deceased, times since loss in months, and cause of death (i.e., COVID-19 and COVID-19 related complication). Moreover, unexpectedness of death, traumatic level of loss, closeness with deceased, and conflict with deceased were measured by single items with a 5-point Likert scale from 1 (not at all) to 5 (very much).

#### International ICD-11 prolonged grief disorder scale (IPGDS) [39]

The IPGDS was developed for assessing symptoms of PGD in ICD-11. The standard scale of the IPGDS contains 13 items about the yearning, preoccupation, emotional distress, and functioning impairment after the death of a close person, and one cultural screening item. Participants indicated how often they experienced these symptoms in the past month on a 5-point Likert scale from 1 (almost never) to 5 (always). A total score of all items excluding the cultural screening item represents the symptom levels of PGD, with higher scores indicating higher levels of symptom. PGD strict criteria were adopted to estimate the prevalence rates: one or two of items 1 or 2, 1 or more of items 3–12, and the impairment criterion (item 13) all rated 4 or above [40]. The IPGDS was validated in both Chinese and German-speaking samples, and the 13 core IPGDS items revealed a two-factor structure that accounted for 57.9% of the variance and received reliability of 0.93 in the Chinese sample [39]. In the current sample, the 13-item IPGDS also resulted in a two-factor structure that explained 53.3% of the variance, and the Cronbach’s alpha was 0.89.

#### Traumatic grief inventory self report (TGI-SR) [41, 42]

The full version of TGI-SR was administered, and the 17-item version of TGI-SR was used for data analysis as it was developed to assess symptoms of PCBD in DSM-5, including yearning, preoccupation, reactive distress to the death, social/identity disruption, and functioning impairment. Participants indicated how often they experienced these symptoms in the past month on a 5-point Likert scale from 1 (never) to 5 (always). A total score of all 17 items represents the symptom levels of PCBD, with higher scores indicating more severe symptoms. The provisional PCBD diagnosis can be made by treating each item rated as 4 or 5 as a symptom endorsed and then follow the DSM-5 based diagnostic rule, which requires endorsement of (a) at least 1 Criterion B item, (b) at least 6 Criterion C items, and (c) the Criterion D item [41, 42]. Since items of the TGI-SR were from the Inventory of Complicated Grief (ICG) [43], the Inventory Complicated Grief-Revised (ICG-R) [44], and the DSM-5 descriptions of PCBD symptoms [5], and the two inventories and the DSM-5 manual were validated in the Chinese samples [45, 46], the content validity of the Chinese version of the TGI-SR should be sufficient. Similar to its original version [41, 42], the scree plots indicated that one-factor solutions accounting for 40.05% of the variance adequately represented the data of the current sample, and factor loadings of all items were larger than 0.40. The Cronbach’s alpha of the 17-item TGI-SR was 0.95 in the bereaved patient sample [41], 0.90 in the disaster-bereaved sample [42], and 0.91 in the current sample.

### Statistical analyses

The characteristics of the sample were summarized using descriptive statistics. Cronbach’s alpha coefficients measured the internal reliability of the IPGDS and the TGI-SR; a cut-off of .70 was used to indicate good internal reliability. Cohen’s kappa was calculated to assess the consistency of screening positive cases by IPGDS and TGI-SR. Multiple linear regression analyses were performed to examine the independent associations between demographic and loss-related characteristics and grief symptoms. The associations were reported as or unstandardized coefficient B, standardized coefficient beta, and 95% confidence intervals (CIs). *P* values < 0.05 were considered statistically significant (two-sided). Analyses were performed using IBM SPSS Statistics 26.

## Results

### Sample characteristics

Table 1 summarized the demographic and loss-related characteristics of the sample. Participants were 32.73 ± 9.31 years old, ranging from 18 to 73. Men accounted for 55.5% of the sample. Most of the participants had received higher education (79.4%) and no religious belief (93.6%). Participants who had lost their partner consisted of 32.9% of the sample, followed by those who lost a parent (23.0%), a grandparent (16.4%), and a friend (15.2%). Participants experienced the death 5.10 ± 1.72 months ago. In general, participants were unexpected for the death, perceived the loss as traumatic, and close to the deceased.

**Table 1:** Demographic and loss-related information (*N* = 422)

Variable	M / n	SD / %
**Age**	32.73	9.31
**Sex**		
Male	234	55.5%
Female	188	44.5%
**Education**		
Junior secondary school and/or below	21	5.0%
Senior secondary school	66	15.6%
College	320	75.8%
Postgraduate and/or above	15	3.6%
**Religious belief**		
No	395	93.6%
Yes^a^	27	6.4%
**Role of deceased**		
Partner	139	32.9%
Child	24	5.7%
Parent	97	23.0%
Grandparent	69	16.4%
Relative^b^	22	5.2%
Friend	64	15.2%
Other^c^	7	1.7%
**Age of deceased**	47.81	21.55
**Time since loss in months**	5.10	1.72
**Cause of death**		
COVID-19	408	96.7%
COVID-19 related complication^d^	14	3.3%
**Unexpectedness of death**	3.67	1.23
**Traumatic level of loss**	3.86	0.97
**Closeness with deceased**	4.15	0.88
**Conflict with deceased**	1.76	1.11
**PGD symptom levels**	41.58	9.60
< 6 months post-loss^e^	41.37	9.61
≥ 6 months post-loss^f^	41.85	9.61
**PCBD symptom levels**	54.07	12.28
< 6 months post-loss^e^	53.64	12.36
≥ 6 months post-loss^f^	54.61	12.19

Note. *M* Mean, *SD* Standard Deviation, *PGD* Prolonged Grief Disorder, *PCBD* Persistent Complex Bereavement Disorder

^a^ Religious belief included Buddhism (*n* = 17), Taoism (*n* = 3), Catholicism (*n* = 3), Christianism (*n* = 3), and Islamism (*n* = 1)

^b^ Relative included uncle (*n* = 5), aunt (*n* = 4), cousin (*n* = 4), grandaunt (*n* = 3), granduncle (*n* = 1), great grandmother (*n* = 1), and not specified (*n* = 4)

^c^ Other relationship included colleagues (*n* = 4), acquaintance (*n* = 2), and not specified (*n* = 1)

^d^ COVID-19 related complication included heart disease (*n* = 2), fever (*n* = 2), acute respiratory distress syndrome (*n* = 1), asthma (*n* = 1), cardiovascular and cerebrovascular diseases (*n* = 1), chronic obstructive pulmonary disease (*n* = 1), diabetes (*n* = 1), high blood pressure (*n* = 1), liver cancer (*n* = 1), lung cancer (*n* = 1), obesity (*n* = 1), and respiratory failure (*n* = 1)

^e^ *n* = 234

^f^ *n* = 188

### Prevalence and symptom levels of PGD and PCBD

The mean score of PGD symptoms was 41.58 ± 9.60 and varied between 18 and 60. The average score of PCBD symptoms was 54.07 ± 12.28 and ranged from 22 to 78. Notably, the grief symptom levels did not differ between participants who bereaved 6 months ago and the rest on either PGD symptoms [t (420) = 0.51, *p* = 0.61] or PCBD symptoms [t (420) = 0.81, *p* = 0.42]. Means and standard deviations of PGD and PCBD symptoms are presented in Table 1.

Participants whose close ones died 6 months ago (*n* = 188) formed a subsample to approximate the prevalence rates of PGD and PCBD. Seventy-one (37.8%) of this subsample met the PGD criteria and 55 (29.3%) met the PCBD criteria. There were 18 participants who met the PGD criteria but not the PCBD criteria, and two participants met the PCBD criteria but not the PGD criteria, kappa = 0.76 ± 0.05, *p* < 0.001, indicating the two diagnostic algorithms were significantly consistent. Furthermore, 69 (36.7%) participants indicated that “*my grief would be considered worse (e.g., more intense, severe and/or of longer duration) than for others from my community or culture*”, yet only half of them met the PGD criteria (*n* = 38) and the PCBD criteria (*n* = 33).

### Correlates of PGD and PCBD symptoms

To investigate which factor correlates with PGD and PCBD symptom the most, multiple linear regression analyses were conducted, with all demographic and loss-related variables were entered as independent variables. The model for PGD was significant, F (18, 403) = 6.72, *p* < 0.001, and explained 23.1% of the variance. The model for PCBD was also significant, F (18, 403) = 7.02, *p* < 0.001, and explained 23.9% of the variance. Both regression models identified that COVID-19 rather than its complications as the cause of death, losing a partner, child, and parent, traumatic levels of the loss, and closeness with the deceased as associated factors of grief symptoms (see Table 2). Moreover, losing a grandparent and conflicts with the deceased were related to PCBD symptoms.

**Table 2:** Regression analyses of PGD and PCBD

	PGD				PCBD			
Variable	B (SE)	95% CI	β	t	B (SE)	95% CI	β	t
Age	−0.10 (0.07)	− 0.25, 0.04	− 0.10	− 1.40	− 0.15 (0.09)	− 0.33, 0.04	−0.11	− 1.55
Sex	0.52 (0.89)	− 1.23, 2.27	0.03	0.58	0.99 (1.13)	−1.24, 3.22	0.04	0.87
Education^a^								
Senior high school	0.88 (2.19)	−3.43, 5.19	0.03	0.40	−0.11 (2.79)	−5.59, 5.37	− 0.003	− 0.39
College	−1.72 (2.05)	− 5.75, 2.30	− 0.08	− 0.84	− 2.68 (2.61)	− 7.80, 2.45	− 0.09	− 1.03
Postgraduate	−1.69 (2.99)	−7.57, 4.19	− 0.03	− 0.57	−2.32 (3.81)	−9.80, 5.16	−0.04	− 0.61
Religious belief^b^	2.15 (1.87)	− 1.53, 5.83	0.06	1.15	2.78 (2.38)	−1.90, 7.47	0.06	1.17
Age of deceased	−0.003 (0.05)	−0.11, 0.10	− 0.01	−0.06	− 0.01 (0.07)	−0.15, 0.12	− 0.02	−1.17
Time since loss in months	0.05 (0.25)	−0.45, 0.55	0.01	0.18	0.11 (0.32)	−0.52, 0.75	0.02	0.35
Cause of death^c^	5.35 (2.45)	0.54, 10.05	0.10	2.19*	7.77 (3.12)	1.64, 13.89	0.11	2.49*
Role of deceased^d^								
Partner	7.80 (2.32)	3.24, 12.37	0.38	3.36**	10.73 (2.95)	4.92, 16.54	0.41	3.63***
Child	8.15 (3.62)	1.03, 15.26	0.20	2.25*	10.27 (4.61)	1.21, 19.32	0.19	2.23*
Parent	5.49 (2.03)	1.49, 9.48	0.24	2.70**	8.39 (2.59)	3.30, 13.47	0.29	3.24**
Grandparent	4.46 (2.43)	−0.33, 9.24	0.17	1.83	6.62 (2.59)	0.53, 12.71	0.20	2.14*
Friend	3.08 (2.34)	−1.52, 7.68	0.12	1.32	4.84 (2.98)	−1.01, 10.69	0.14	1.63
Unexpectedness of death	−0.07 (0.39)	−0.83, 0.70	− 0.009	−0.17	− 0.09 (0.49)	− 1.06, 0.88	−0.009	− 0.19
Traumatic level of loss	1.71 (0.61)	0.52, 2.90	0.17	2.82**	2.23 (0.77)	0.71, 3.74	0.18	2.89**
Closeness with deceased	1.60 (0.64)	0.34, 2.86	0.14	2.50*	1.72 (0.81)	0.12, 3.32	0.12	2.11*
Conflict with deceased	0.57 (0.42)	−0.26, 1.40	0.07	1.34	1.05 (0.54)	−0.008, 2.11	0.10	1.95†

Note. *PGD* Prolonged Grief Disorder, *PCBD* Persistent Complex Bereavement Disorder, *SE* Standard Error. *CI* Confidence Interval

^a^ reference group: junior high school and below

^b^ reference group: no religious belief

^c^ reference group: COVID-19 related complication

^d^ reference group: a combined group of relative and other relationships

† *p* = 0.05, * *p* < 0.05, ** *p* < 0.01, *** *p* < 0.001

## Discussion

This is the first study to estimate the prevalence of PGD in ICD-11 and PCBD in DSM-5 and to identify demographic and loss-related factors associated with PGD and PCBD symptoms in a large sample of individuals bereaved due to COVID-19. Using the latest diagnostic criteria of pathological grief in ICD-11 and DSM-5 and the most updated measures for PGD and PCBD, this study demonstrates that over one-third of COVID-19 related bereaved individuals suffered from PGD or PCBD. Factors associated with more severe grief symptoms were losing a close person by COVID-19 rather than related complications, losing a partner, child, parent, grandparent, feeling more traumatic about the loss, being closer to the deceased, and having more conflicts with the deceased.

Prevalence of PGD found in our COVID-19 related bereaved sample (37.8%) is higher than Chinese people bereaved 55 months ago (12.7%) and German-speaking people bereaved 48 months ago (7.3%); both studies used the same measure and diagnostic algorithm [39]. The number is also higher than the prevalence of PGD in a sample of bereaved Wenchuan earthquake survivors in China (8.47%) [16] and a group of Chinese *Shidu* parents (i.e., parents who lost their only child) bereaved 7 years ago (22.2%) [47], who are undergoing the most painful and traumatic experience than other types of loss and facing physical and psychological challenges [48]. The number is similar to the prevalence in a large-scale survey of Chinese *Shidu* parents bereaved 9 years ago (35.5%) [49]. Except for the prevalence, the symptom levels of PGD (41.85 ± 9.61) were higher than those reported in the German-speaking sample (29.22 ± 10.83) and the Chinese sample (36.29 ± 11.35).

Rate of PCBD in our study (29.3%) is higher than that in two Dutch bereaved patient samples, namely 17.7% [41] and 12.5% [42]. The number is also higher than that in Dutch citizens bereaved 344 days ago due to the Ukrainian plane disaster (6.6%) [42]. While the prevalence of PCBD in our sample is higher than in previous studies, the symptom levels measured by the 18-item version of TGI-SR (57.39 ± 13.10) seems equivalent to that reported among 49 Dutch people bereaved about 2 months ago due to COVID-19 (57.37 ± 9.60) [26].

Not surprisingly, time since loss may help to explain the high prevalence of PGD and PCBD in the COVID-19 bereaved population as the survey was conducted approximately 9 months since the first COVID-19 case reported in China, which was less than all of the previous studies. Nevertheless, while the time frame of the survey could be partially accounted for the relatively high prevalence of PGD and PCBD in the COVID-19 bereaved population, our findings still support concerns from researchers that there would be a rise in PGD [8, 9] because acute grief is among the strongest predictors of the development of PGD [50]. The finding that no difference of grief symptoms was detected between participants bereaved 6 months ago or less than 6 months echoes in part that acute grief might greatly correlate with prolonged grief, as the time criterion for PGD in ICD-11 was at least 6 months [4].

These findings add to the discussion on the PGD and PCBD criteria in terms of the symptom criteria and the time criterion. The first question is that whether the symptom criteria should be stricter for unnatural death as grief reactions for unnatural death might be more severe in nature, as shown in this study and another study on deaths due to the COVID-19 pandemic [26]. Second, as severe grief reactions last for more than 6 months in the context of the pandemic, which may imply that the duration of acute grief for mass bereavement could be longer, the time criterion in ICD-11 for diagnosing PGD (i.e., at least 6 months) remains questionable. Therefore, some scholars are advocating for extending the time criterion to 12 months [51]. On the other hand, however, it is possible that under the circumstance of mass bereavement, a shortened time criterion would facilitate providing timely help for those in need.

Another interesting point is that over one-third of participants who bereaved for more than 6 months believed their grief reactions went beyond cultural norms, yet only half of them met the symptom and functioning criteria. This reflects a discrepancy in the understanding of “abnormal grief” between the bereaved and professionals: in the case of bereavement experience due to COVID-19, some bereaved individuals tended to believe that their grief went wrong even though their grief was assessed to be within the normal range in terms of both severity and functioning impairment from the professionals’ perspective. Efforts should be made to narrow the gap of understanding of pathological grief and to seek consensus between the bereaved and the professionals regarding the consideration for social, cultural, or religious norms [52].

Although the diagnostic algorithms of PGD and PCBD in this study were generally consistent, prevlance of PCBD (29.3%) was lower than that of PGD (37.8%), suggesting that the DSM-5 PCBD criteria may be more strict. A previous study compared six proposed diagnostic criteria sets for disturbed grief and also found lower prevalence of DSM-5 PCBD (11.1%) than ICD-11 PGD (19.8%) [7]. The inconsistency between the two diagnoses was believed to bring negative impact on research and care [53], and debate on which diagnosis is more clinical useful is ongoing. Some found that hightening the threshold of ICD-11 PGD criteria could increase its agreement with DSM-5 PCBD [53], whereas the others showed that ICD-11 PGD symptoms were better-captured psychopathology, more sensitive to change over time [54], and more responsive to treatment [55].

Most of the associated factors identified in this study are consistent with previous research. First, verifying our hypothesis, those who grieve the most were the ones who lost a first-degree family member (i.e., partner, parent, child), followed by grandparents (a significant correlate for PCBD symptoms), friends, relatives, and other relationships. This pattern strengthens the importance of considering the kinship between the bereaved and the deceased across causes of death [13, 16, 27–34]. Second, although all participants lost their close ones due to COVID-19, a handful of them endorsed COVID-19 induced complications as the causes of death and they experienced less severe grief symptoms than their peers. Despite the fact that deaths during the pandemic were generally unexpected, since those who died from complications was older [59.14 ± 16.79 versus 47.42 ± 21.61, t (420) = 2.008, *p* = 0.045] and usually had a history of diseases, the bereaved might be more mentally prepared for the death than others, and preparedness for death served as a protective factor for grief symptoms [35, 56].

We found that in the grieving process during the pandemic, subjective traumatic level of the loss was more crucial than unexpectedness of the death in association to grief symptoms, which partially supports our hypotheses. Compared to the objective traumatic deaths (e.g., violent deaths), subjective experience of death as traumatic was a more significant factor that contributing to grief symptoms [18]. During the COVID-19 pandemic, because of the nationwide lockdown measures taken to prevent the spread of the virus in China, people were unable to celebrate the Spring Festival by gathering together in late January, hold funerals and farewell rituals for the deceased, offer condolences to bereaved families by physical company, and memorialize decedents by sweeping the tomb on the Tsing Ming Festival in April. Additionally, owning to the contagious nature of the pandemic, family members were not allowed to say goodbye to the dying patient, let alone keeping some meaningful personal belongings of the deceased [57]. All these created obstacles to the grieving process, which may result in prolonged grief.

Confirming our hypothesis, quality of relationship between the bereaved and the deceased also plays a part concerning grief severity and our findings suggest that assessing the ambivalence in the relationship is necessary as both closeness and conflict with the deceased were positively correlated with grief severity. The closer to the deceased before the death, the stronger attachment was established with the deceased. The bond of attachment would continue even when they are separated by death, yet it might imply disbelief that the person is dead and thus lead to unresolved grief [58]. Death of an attachment figure presents a “temporarily irreconcilable mismatch between an unrevised mental representation of a loved one and a dramatic change in the ongoing relationship with that person” (p.454), resulting in acute grief symptoms such as yearning for the deceased, preoccupation of the deceased, and loss of interest in the world [59]. Once the mental representation of the deceased was revised by incorporating the reality of death, acute grief symptoms would be resolved [37]. However, for individuals who are deeply attached to the deceased, the revision of the mental representation could take a longer time, and thus manifested as prolonged grief symptoms. Another finding we would like to highlight is that more conflicts with the deceased before the death, although at a smaller magnitude than closeness, is related to more severe PCBD symptom. It is the first study, to our knowledge, to provide empirical evidence for the relationship between conflict with the deceased and mental health. Conflicts existed at the time of death and earlier are assumed to be accompanied by a strong psychological dependence on the deceased [38], and greater dependency is a risk factor for prolonged grief symptoms [60].

This study contributes to the clinical practice for bereaved people in several aspects. First, as our findings confirmed the concerns about the rise of PGD after the outbreak of COVID-19 and the pandemic tends to be sustained, mental health practitioners need to be alert when caring for the bereaved and assess whether and when their grief goes pathological. Second, when assessing acute and prolonged grief of bereaved people, practitioners need to be aware of the difference between the ICD-11 and DSM-5 diagnostic guidelines for pathological grief and both guidelines should be considered in order to avoid omitting those in need. Third, not only those who had lost their first-degree relatives, but also those who had lost their grandparents and friends need to be included when providing bereavement care. Fourth, it is important to understand how the COVID-19 pandemic had changed the bereaved people’s perception of the death and their experience of the grieving process, as the subjective traumatic levels of the loss served a significant correlates to elevated grief symptoms. Last, quality of the relationship between the deceased and the bereaved is worth exploring, and both positive and negative memories should be examined to resolve the unfinished business and thus to facilitate a more adaptive grieving process.

This study also has limitations. First, the sample is recruited by convenience sampling, whose representativeness may be affected by the self-selection bias. The current sample might experience less severe grief symptoms than a random sample, as it may be more taxing for those who suffered from more severe grief symptoms to fill out all questions. Thus, the study may underestimate the severity of PGD and PCBD symptoms among COVID-19 bereaved individuals. Second, although strictly following the ICD-11 and DSM-5 diagnostic guidelines, this study adopted self-reported measures rather than structured clinical interviews to determine the prevalence rates. Similarly, data on whether the death was due to COVID-19 was reported by the participants rather than extracted medical records. Third, due to the cross-sectional design, effects of demographic and loss-related variables in predicting the development of PGD and PCBD could not be examined. Longitudinal study is needed to determine the causality. Last, loss-related characteristics specifically affected by the COVID-19 pandemic was not included in explaining the variance of grief symptoms. Future research can investigate the relationship between grieving experience altered by COVID-19 and the development of PGD and PCBD so that bereavement support could be tuned for the COVID-19- or pandemic-related bereaved population.

Notwithstanding these considerations, the study contributes to the field by providing the first evidence of the prevalence, symptom severity, and associated factors of PGD and PCBD in a sample of COVID-19 bereaved individuals. It compared the prevalence of pathological grief between ICD-11 and DSM-5 in a Chinese population, adding cross-cultural data of adopting these two diagnostic systems in the midst of utilizing and revising the diagnostic criteria [7, 61].

## Conclusions

The prevalence of PGD in COVID-19 bereaved people was 37.8% and that of PCBD was 39.9%. Factors associated with more severe PGD and PCBD symptoms were losing a close person by COVID-19 rather than complications, losing a partner, child, parent, grandparent, feeling more traumatic about the loss, being closer to the deceased, and having more conflicts with the deceased. The high prevalence found in this study validates concerns and early interventions for people bereaved due to COVID-19, and a targeted group that requires more support could be identified based on the associated factors reported in this study.

## Data Availability

The datasets used and/or analysed during the current study are available from the corresponding author on reasonable request.
